# The World’s Exposition

**Published:** 1885-04

**Authors:** 


### THE WORLD'S EXPOSITION.

BY MRS. M. W. J.

The opening of the World's Exposition marks an epoch in the history of the nations of the earth.

Here may be traced, in chronological order, the workings of the mind, the development of the genius, the results of the handiwork of man, from the very infancy of the human race, onward to the grandest achievements of the most advanced scientific minds of the present day.

The physical development of man may be traced from Ward's cast of the Neanderthal man—probably the oldest existing relic of prehistoric man, contemporaneous with the fossil mammoth of the Rhine valley—through skeletons and skulls and casts of the brains of men of all ages and all races.

In addition to Ward's celebrated series, there is in the Missouri section a very rich collection of archæological specimens and Indian antiquities; also some fossil remains, embracing a number of human skeletons dug from the ancient mounds. This collection, which is contributed by Prof. Broadhead, of St. Louis, is very interesting and valuable.

The intellectual progress of man, as evidenced in his handiwork, may be traced from the rude homes of the ancient cave and cliff and mound dwellers, as seen in the Smithsonian reproductions in the exhibit, on to models of the most modern structures of dwellings, churches, hospitals, etc.

The development of "the inner and the outer man," as evidenced by the provisions made for his gradually increasing wants and requirements, from the bare necessities of animal life on to the æsthetic luxuries of the present day, may be studied through the primitive hunting and fishing implements, rude pottery and basket ware, remnants of bark and skin garments, which have been unearthed from among the ruins of those ancient dwellings, and which sufficed to supply the needs of primeval man, down to the exquisite Venetian and Bohemian glass-ware and the translucent porcelain, or the gorgeous fabrics of the modern loom, the rich silks and velvets, the dainty gossamers, the "purple and fine linen" which minister to the refinements of our highest civilization.

All that mankind has learned, from the beginning of time to the present day, in manufactures, in inventions, in the products of genius or of skill, all that is beautiful in art or wonderful in science, all that the world holds valuable, whether as the production of the earth or as the invention of man, may here be seen in its consummated form. The whole earth has poured her treasures into the lap of the Exposition.

The exhibits of the national department of state form a complete technological school, illustrated by contributions forwarded by the U. S. consuls in all parts of the world, the geography and the history, the industries and the specialties of every nation on the globe being thus set forth in one grand tableau.

In all the crowds that throng the vast buildings or wander through the extensive grounds, there cannot be found a man, woman or child, who will not find somewhere within the enclosure the one thing of all others that he has most wished to see or study.

No matter what may be his calling, or where his home, he will find that which has hitherto seemed most inaccessible or unattainable.

The farmer will find samples of the soils of all lands, the products of all soils and all climates.

The sailor will find models of the most famous vessels that have ever been constructed. The painter and the sculptor will find the rarest gems of art.

The musician will find instruments of every age and every make, and can listen to strains of classic music interpreted by the most skillful musicians.

The student of the natural sciences will find, in the Smithsonian exhibit or in Ward's collection, his own special branch of study most fully illustrated, in systematic cabinets devoted to mineralogy, geology, paleontology, zoology, botany, taxidermy, etc.

The educational exhibit is very complete.

The medical student will find a fine display from the department of medicine and surgery of the U. S. Navy—models of army hospitals, floating hospitals, travelling hospitals, models of wounds and scars and amputations, artificial limbs, exhibits of sanitary arrangements for the sick on board men-of-war, etc. There is also a model of a crematory, constructed of iron, and in perfect working order.

Prof. Nissen, of Sweden, exhibits his system of gymnastic apparatus, for correcting physical defects and developing the muscular system.

Caswell, Hazard & Co., of New York, show a complete hospital outfit, embracing every possible requisite, while Seabury and Johnson show all kinds of medicinal plasters.

The displays of drugs and chemicals, salts and crystals, are showy and elegant in their bright colors and prismatic tints, forming castles and grottoes and pyramids, sparkling and glowing under the electric lights.

The Dental exhibit is rather limited, the S. S. White Co. having the only large and elaborate display. This is most tastefully arranged, the furniture consisting of their handsomest dental chairs and attachments, bracket-tables, fountain-spittoons, etc. Their instruments of every variety, as also teeth and plate-work, are handsomely arranged, gleaming against a background of dark velvet. Handsome silver-mounted show-cases also display a full line of their various "specialties for the mouth," washes, pastes, powders, tablets, etc.

In the Iowa exhibit of woman's work is a display of platework by the first female dentist licensed in that State.

J. H. Meyer, of 159 W. 34th street, New York, has a very tasteful display of platework.

G. Sibley has also a neat case of teeth.

On a pedestal bordered with elegant lambrequins, Vail Brothers, of Philadelphia, have built a lofty pyramid composed entirely of bottles of their very superior tooth-powder. They also present every purchaser with an elegant *souvenir* of the Exposition.

In the foreign exhibits, A. Chonet et Cie, of Paris, display the tooth-wash and the quinquina and coral tooth-powders of Docteur Pierre.

In the Russian exhibit is an elegant display of gold leaf and foil, from Smirnoff, of Moscow; also a case of platework medals and crosses from Dr. L. Bernardo-Berkaneer, of the same city.

In the Mexican department may be seen, in a glass case, a curious branching cactus plant, bearing a remarkable crop of flowers in the shape of numerous medals bestowed upon Dr. Antonio Roque, "Chief Director of the Surgical-Dentistry Department of Public Assistance," the fruit of the same being a meagre assortment of rubber and celluloid platework.

Another glass case in the same exhibit contains an assortment of dental instruments, with the card of Dr. R. Lavista.

There is also platework from the office of Dr. Jose-Maria Soriano, who has apparently associated with himself the American dentists, as the exhibit also bears the names of Drs. Keller, Thompson, and Wyse. This concludes the list of all the dental displays that I have been able to find in the World's Exposition, but there is enough here to interest every dental observer.

